# Syphilis Trends among Men Who Have Sex with Men in the United States and Western Europe: A Systematic Review of Trend Studies Published between 2004 and 2015

**DOI:** 10.1371/journal.pone.0159309

**Published:** 2016-07-22

**Authors:** Winston E. Abara, Kristen L. Hess, Robyn Neblett Fanfair, Kyle T. Bernstein, Gabriela Paz-Bailey

**Affiliations:** 1 Division of HIV/AIDS Prevention, National Center for HIV, Hepatitis, STD and TB Prevention, Centers for Disease Control and Prevention, Atlanta, Georgia; 2 Division of STD Prevention, National Center for HIV, Hepatitis, STD and TB Prevention, Centers for Disease Control and Prevention, Atlanta, Georgia; British Columbia Centre for Excellence in HIV/AIDS, CANADA

## Abstract

Globally, men who have sex with men (MSM) are disproportionately burdened with syphilis. This review describes the published literature on trends in syphilis infections among MSM in the US and Western Europe from 1998, the period with the fewest syphilis infections in both geographical areas, onwards. We also describe disparities in syphilis trends among various sub-populations of MSM. We searched electronic databases (Medline, Embase, Global Health, PsychInfo, CAB Abstracts, CINAHL, Sociological Abstracts, Web of Science, Cochrane Library, and LILACS) for peer-reviewed journal articles that were published between January 2004 and June 2015 and reported on syphilis cases among MSM at multiple time points from 1998 onwards. Ten articles (12 syphilis trend studies/reports) from the US and eight articles (12 syphilis trend studies/reports) from Western Europe were identified and included in this review. Taken together, our findings indicate an increase in the numbers and rates (per 100,000) of syphilis infections among MSM in the US and Western Europe since 1998. Disparities in the syphilis trends among MSM were also noted, with greater increases observed among HIV-positive MSM than HIV-negative MSM in both the US and Western Europe. In the US, racial minority MSM and MSM between 20 and 29 years accounted for the greatest increases in syphilis infections over time whereas White MSM accounted for most syphilis infections over time in Western Europe. Multiple strategies, including strengthening and targeting current syphilis screening and testing programs, and the prompt treatment of syphilis cases are warranted to address the increase in syphilis infections among all MSM in the US and Western Europe, but particularly among HIV-infected MSM, racial minority MSM, and young MSM in the US.

## Introduction

Globally, men who have sex with men (MSM) account for a disproportionate burden of syphilis infections [1–3]. In the US, the prevalence rates of primary and secondary syphilis are highest among MSM, particularly among young and minority MSM [2]. In Western Europe, MSM also account for the majority of primary and secondary syphilis cases and remain the group most at risk for contracting syphilis [3]. Primary and secondary syphilis are the most infectious stages of syphilis [4]. Syphilis causes inflammatory genital ulcers and lesions which can increase the risk of HIV transmission by increasing HIV shedding, and acquisition by providing a portal of entry to the HIV virus [5]. Syphilis also complicates the clinical course of HIV by increasing viral load [5, 6]. It has also been associated with a higher rate of treatment failure in HIV-infected persons [7].

The HIV/AIDS epidemic in the 1980s was associated with general declines in the rates of syphilis among the general population in the US and Western Europe, including MSM [1, 8, 9]. The decline continued into the early 1990s and was concurrent with the adoption of safer sex behaviors and the effectiveness of sexual education and HIV/AIDS prevention programs [10, 11]. However, around 1999, intermittent outbreaks of syphilis were reported in many urban areas in the US and Western Europe [8–13]. These outbreaks occurred almost exclusively among MSM and were attributed to increases in risky sexual behaviors such as condomless anal sex (CAS), exchange sex, illicit drug use before sex, multiple sexual partners, and high-risk anonymous sexual contacts [14]. Syphilis outbreaks continue to occur sporadically in the US and Western Europe [3].

Given the continued risk of syphilis transmission, its close association with HIV infection, and the disproportionate disease burden among MSM in the US and Western Europe, there is a need to examine and understand syphilis trends among MSM in both geographic areas. The purpose of this systematic review is to descriptively examine and compare recent trends over time in syphilis cases among MSM in the US and Western Europe. This review will also describe disparities in syphilis trends by HIV status, race, and age among MSM with the aim of identifying sub-groups that would benefit from enhanced syphilis screening and intervention strategies in the US and Western Europe. The current review focuses on syphilis data from 1998, the year record lows in the rate of syphilis cases in the US and Western Europe were reported, and afterwards [9, 15].

## Methods

### Database and search strategy

A systematic literature search was conducted to find research papers and surveillance reports that assessed changes over time (1998–2015) in sexual risk behaviors and the prevalence of sexually transmitted infections (STI) among MSM. A Centers for Disease Control and Prevention librarian conducted a search of multiple electronic databases (Medline, Embase, Global Health, PsychInfo, CAB Abstracts, CINAHL, Sociological Abstracts, Web of Science, Cochrane Library, and LILACS) by cross-referencing multiple search terms in three domains, 1) sexual risk behavior/STI descriptors (sexual behavior, safe sex, protected sex, unsafe sex, unprotected sex, anal intercourse, condom use, high-risk sex, bare backing, sex partner, sex risk, sexually transmitted disease, STD, sexually transmitted infection, STI, syphilis, gonorrhea, chlamydia, lymphogranuloma venereum), 2) MSM descriptors (homosexuality, men who have sex with men, MSM, men having sex with men, gay men, bisexual men, homosexual) and 3) trend descriptors (trend, pattern, change, increase, decrease, stable, unchanged, over time, epidemiology, survey, surveillance). Articles for inclusion in this review were further limited to studies and surveillance reports that met the following criteria: reported on MSM who resided in the US or Western Europe; reported on syphilis (primary, secondary, and latent) cases at multiple time points with dates of onset, diagnosis, or official notification to a public health authority from 1998 to 2015; reported in full length peer-reviewed publication (abstracts, posters, books, and dissertations were excluded); and published in English from January 2004 to June 2015. In summary, for this review, we limited eligible articles to peer-reviewed studies and surveillance reports on syphilis trend data on MSM in the US and Western Europe that commenced from 1998 or afterwards, and were published in English between 2004 and 2015. Eligible studies were limited to those published between 2004 and 2015 in order to review recently published studies and minimize the inclusion of multiple studies with syphilis trends data over the same time period. Modeling studies, intervention studies, systematic reviews, and meta-analyses were excluded. We did not include data that was obtained exclusively from institutionalized populations and very high-risk populations such as sex workers because data from these populations may influence the interpretation of our findings. For the purpose of this study, Western Europe refers to countries that are part of the United Nations Regional Centre for Western Europe [16].

### Data abstraction

Data were coded for location of residence of cases (country and city), time frame (i.e., years syphilis diagnosis, case, or surveillance data were collected), recruitment and sampling methodology (i.e., surveillance, periodic survey of probability, or convenience sample), overall syphilis trends (increase, decrease, or stable), syphilis trends by sample characteristics (HIV status, race, age), statistical significance testing, outcome measure (syphilis case numbers and syphilis case rates), and stage of syphilis. We extracted syphilis trend data commencing in 1998 or later from all studies and reports. If an article presented unduplicated syphilis trend data from more than one city or country separately, we presented trend data from these cities or countries separately in the tables and graphs, otherwise aggregate results were presented. A two-tailed level of significance value of 0.05 or less was considered significant for studies that reported tests of significance for trend data. Data were extracted independently and then reviewed by two people to ensure their accuracy. This systematic review and the methodological evaluation of each study were written up according to the PRISMA standard (a protocol used to evaluate systematic reviews) (S1 Checklist) [17].

## Results

**Fig 1:**
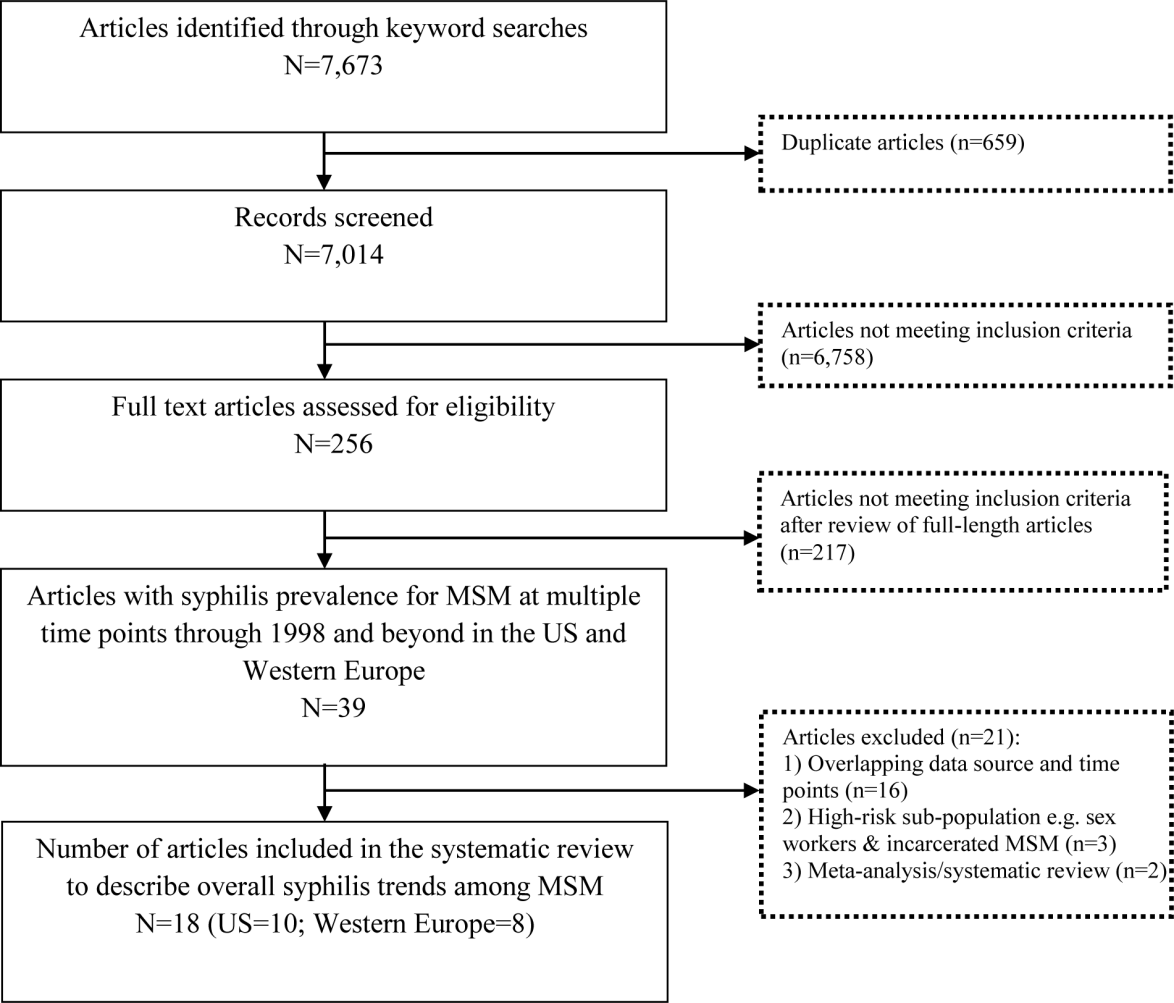
Flow diagram showing the article screening process.

Fig 1 is a flow diagram showing the article screening process. The initial search resulted in 7,673 citations. After removing duplicates, there were 7,014 citations to review for inclusion. Of these, 6,758 citations did not meet the inclusion criteria and 256 citations were retained for retrieval of the full-length article. Review of the full-length articles yielded 39 articles that met the inclusion criteria. If multiple articles reported on overlapping data sources or time points, the most recent or most comprehensive article (larger sample size, more time points, trend data showing disparities by HIV status, race, or age, etc.) was included. Through this process, 16 articles were excluded because they contained duplicate data and outcomes. An additional five articles were excluded because the data were specific to a very high-risk sub-population (i.e., sex workers and incarcerated MSM; n = 3), or because the article described a meta-analysis (n = 2). A final list of 18 articles was used to describe overall syphilis trends for this systematic review. These 18 articles (10 from the US and 8 from Western Europe) included data from independent convenience samples and case surveillance reports from different cities and countries that were considered in this paper to be 24 unduplicated studies.

### General description of syphilis trend studies conducted in the US and Western Europe

Of the 18 articles (24 studies) that met the inclusion criteria, 10 articles (12 studies) reported overall syphilis trends among MSM who lived in the US [18–27] and 8 articles (12 studies) reported overall syphilis trends among MSM who lived in Western Europe [28–35]. One article from the US included syphilis trend data from three different cities [25], resulting in 12 studies from 10 articles. Similarly, one article from Western Europe included syphilis trend data from five different countries in Western Europe [32], resulting in 12 studies from 8 articles. Table 1 presents a summary of the syphilis trend studies in the US; Table 2 presents a summary of syphilis trend studies in Western Europe. Among the trend studies conducted in the US, four were conducted in California [18–21]; one each was conducted in Alabama [22], Washington [23], and Wisconsin [24]; one study obtained data from three cities (New York City, Miami, and Philadelphia) [25]; one study obtained syphilis case report data from 27 states [26]; and another study utilized syphilis case surveillance data from every state and the District of Columbia that were reported to the CDC [27].

**Table 1:** Syphilis trend studies among MSM conducted in the United States, 2004–2015.

Citation	Location	Years data collected	Sampling method	Syphilis trends (increase, stable, or decrease)	Significance test reported	p-value	Outcome measure	Stage of syphilis
***Studies used to describe overall syphilis trends in the US***								
Bernstein et al, 2013 [18]	San Francisco, California	2001–2011	Case surveillance data	Increase, followed by a decrease and then a period of resurgence	No		Number of cases	Primary and secondary
Biedrzycki et al, 2011 [24]	Milwaukee, Wisconsin	1999–2008	Case surveillance data	Increase	No		Number of cases	Primary and secondary
Brewer et al, 2011 [25]	New York City	2000–2008	Case surveillance data	Consistent and significant increase among all MSM	Yes	p < .05	Number of cases	Primary and secondary
Brewer et al, 2011 [25]	Miami-Fort Lauderdale	2000–2008	Case surveillance data	Consistent and significant increase among older (≥20 years) MSM	Yes	p < .05	Number of cases	Primary and secondary
Brewer et al, 2011 [25]	Philadelphia	2000–2008	Case surveillance data	Consistent and significant increase among older (≥20 years) MSM	Yes	p < .05	Number of cases	Primary and secondary
Chew et al, 2013 [19]	California (excludes San Francisco)	2004–2008	Case surveillance data	Consistent increase	No		Number of cases	Primary and secondary
Dilley et al, 2004 [20]	Los Angeles California	2000–2002	Case surveillance data	Consistent increase	No		Number of cases	Primary and secondary
Gunn et al, 2007 [21]	San Diego, California	1998–2004	Case surveillance data	Consistent increase	No		Number of cases	Primary and secondary
Hook et al, 2009 [22]	Jefferson County, Alabama	2002–2007	Case surveillance data	Increase	No		Number of cases	Primary and secondary
Kerani et al, 2007 [23]	King County, Washington	1998–2005	Case surveillance data	Increase	No		Number of cases	Primary, secondary, and early latent
Peterman et al, 2014 [27]	All the states in the US and the District of Columbia	2009–2013	Case surveillance data	Consistent increase	No		Number of cases	Primary and secondary
Su et al, 2011 [26]	27 states in the US	2005–2008	Case surveillance data	Consistent increase	Yes	p < .05	Rate	Primary and secondary
***Studies used to describe disparities in syphilis trends among MSM by HIV infection in the US***								
Biedrzycki et al, 2011 [24]	Milwaukee, Wisconsin	1999–2008	Case surveillance data	Increasing syphilis trends among HIV-positive MSM	No		Number of cases	Primary and secondary
Kerani et al, 2007 [23]	King County, Washington	1998–2005	Case surveillance data	Increasing syphilis trends among HIV-positive MSM compared to HIV-negative MSM	No		Rate	Primary, secondary, and early latent
***Studies used to describe racial disparities in syphilis trends among MSM in the US***								
Fuqua et al, 2015 [39]	San Francisco, California	2004–2011	Case surveillance data	Increasing syphilis trends among Black MSM	No		Number of cases	All syphilis cases (no stage specified)
McFarland et al, 2004 [38]	San Francisco, California	1999–2002	Case surveillance data	Increasing syphilis trends among Asian/Pacific Islander MSM compared to White MSM	No		Rate	Primary, secondary, and early latent
Patton et al, 2014 [2]	34 states in the US and the District of Columbia	2009–2012	Case surveillance data	Increasing syphilis trends among Hispanic MSM compared to White MSM	No		Number of cases	Primary and secondary
Su et al, 2011 [26]	27 states in the US	2005–2008	Case surveillance data	Increasing syphilis trends among Black and Hispanic MSM compared to White MSM	Yes	p < .05	Rate	Primary and secondary
***Studies used to describe age disparities in syphilis trends among MSM in the US***								
Brewer et al, 2011 [25]	New York City	2000–2008	Case surveillance data	Consistent and significant increase among MSM between 15–19 years, 20–24 years and ≥25 years	Yes	p < .05	Number of cases	Primary and secondary
Brewer et al, 2011 [25]	Miami-Fort Lauderdale	2000–2008	Case surveillance data	Consistent and significant increase among MSM between 20–24 years and ≥25 years	Yes	p < .05	Number of cases	Primary and secondary
Brewer et al, 2011 [25]	Philadelphia	2000–2008	Case surveillance data	Consistent and significant increase among MSM between 20–24 years and ≥25 years	Yes	p < .05	Number of cases	Primary and secondary
Patton et al, 2014 [2]	34 states in the US and the District of Columbia	2009–2012	Case surveillance data	Increasing syphilis trends among MSM between 20 and 29 years compared to other age groups	No		Number of cases	Primary and secondary
Su et al, 2011 [26]	27 states in the US	2005–2008	Case surveillance data	Increasing syphilis trends among MSM between 20 and 24 years compared to other age groups	No		Rate	Primary and secondary

**Table 2:** Syphilis trend studies among MSM conducted in Western Europe, 2004–2015.

Citation	Location	Years data collected	Sampling method	Syphilis trends (increase, stable, or decrease)	Significance test reported	p-value	Outcome measure	Stage of syphilis
***Studies used to describe overall syphilis trends in Western Europe***								
Cowan et al, 2012 [34]	Denmark	1995–2010	Case surveillance data	Increase	No		Number of cases	All syphilis cases (no stage specified)
*ECDC,2012 [32]	France	2000–2010	Case surveillance data	Increase	No		Number of cases	All syphilis cases (no stage specified)
*ECDC,2012 [32]	Sweden	2000–2010	Case surveillance data	Increase	No		Number of cases	All syphilis cases (no stage specified)
*ECDC,2012 [32]	Netherlands	2004–2010	Case surveillance data	Increase	No		Number of cases	All syphilis cases (no stage specified)
*ECDC,2012 [32]	Ireland	2000–2010	Case surveillance data	Increase	No		Number of cases	All syphilis cases (no stage specified)
*ECDC,2012 [32]	Greece	2008–2010	Case surveillance data	Consistent increase	No		Number of cases	All syphilis cases (no stage specified)
Hopkins et al, 2004 [31]	Ireland	1998–2003	Convenience sampling: Data from a health center	Increase	No		Rate	Primary and secondary
Jebbari et al, 2011 [28]	England and Wales	1999–2008	Case surveillance data	Increase	No		Number of cases	Primary, secondary, and early latent
Malek et al, 2015 [29]	England	2009–2013	Case surveillance data	increase	No		Number of cases	Primary, secondary, and early latent
Jakopenac et al, 2010 [33]	Norway	1992–2008	Case surveillance date	Increase	No		Number of cases	Primary, secondary, and early latent
Potts et al, 2014 [30]	Scotland	2005–2013	Case surveillance data	Increase followed by a decrease at the end of the study period	No		Number of cases	Primary, secondary, and early latent
Savage et al, 2009 [35]	Germany	2001–2007	Case surveillance data	Consistent increase	No		Number of cases	Primary and secondary
***Studies used to describe disparities in syphilis trends among MSM by HIV infection in Western Europe***								
Cowan et al, 2012 [34]	Denmark	1995–2010	Case surveillance data	Increasing syphilis trends among HIV-positive MSM	No		Number of cases	All syphilis cases (no stage specified)
Hopkins et al, 2004 [31]	Ireland	1998–2003	Convenience sampling: Data from a health center	Increasing syphilis trends among HIV-positive MSM compared to HIV-negative MSM	No		Rate	Primary and secondary
Jakopenac et al, 2010 [33]	Norway	1992–2008	Case surveillance date	Increasing syphilis trends among HIV-positive MSM	No		Number of cases	Primary, secondary, and early latent
Malek et al, 2015 [29]	England	2009–2013	Case surveillance	Increasing syphilis trends among HIV-positive MSM	No		Rate	Primary, secondary, and early latent
Marcus et al, 2005 [41]	Germany	2001–2004	Case surveillance data	Increasing syphilis trends among HIV-positive MSM	No		Number of cases	All syphilis cases (no stage specified)
***Studies used to describe age disparities in syphilis trends among MSM in Western Europe***								
Koedijk et al, 2014 [37]	Netherlands	2006–2012	Case surveillance data	Decreasing syphilis trends among both younger (15–24 years) and older (≥25 years) MSM	No		Rate	Primary and secondary

*ECDC–European Centre for Disease Prevention and Control

Case surveillance data were used to analyze syphilis trends for all the studies conducted in the US [18–27]. Two studies conducted tests of significance to determine if syphilis case data changed significantly over time [25, 26]. The most common measure used in determining syphilis trends was the number of reported syphilis cases–ten studies evaluated the number of reported syphilis cases over time and two studies reported syphilis case rates. For the two studies that estimated rates, denominators were obtained from US census data and published reports [23] and from the National Center for Health Statistics [26]. Of the 12 studies that reported overall syphilis trends, 11 reported on trends in infectious syphilis (primary and secondary syphilis) and one reported on trends in primary, secondary, and early latent syphilis (Table 1).

Of the 12 studies conducted in Western Europe (see Table 2), one each was conducted in England and Wales [28], England [29], and Scotland [30]. Two studies used data from Ireland (31, 32) while the other studies were each conducted in France [32], Sweden [32], Greece [32], the Netherlands [32], Norway [33], Denmark [34], and Germany [35]. One surveillance report presented comprehensive syphilis surveillance trends among MSM from eight Western European countries [32]. From this comprehensive syphilis surveillance report, we included non-duplicate data from five countries–Ireland, France, Sweden, Greece, and the Netherlands. We excluded data from the other two countries because they duplicated data from studies already included in our review or were not as comprehensive. For 11 of the 12 studies from Western Europe, the investigators obtained data from syphilis case surveillance systems and used the number of syphilis cases to assess syphilis trends [28–30, 32–35]. Among the three studies that reported syphilis case rates, denominators were obtained from general population surveys and census population estimates for MSM [31], a national database of MSM with current HIV diagnoses [29], and the number of syphilis tests among MSM in a national surveillance system [36]. Two studies reported trends in infectious syphilis only, four reported on primary, secondary, and early latent syphilis, and six reported on syphilis diagnoses (no stage specified) (Table 2).

Of the 21 countries in Western Europe, nine countries in Western Europe consistently collect syphilis surveillance data on MSM [32, 35]. This review included data from eight of these countries in Western Europe that met our inclusion criteria. Overall, we included both national surveillance report (national data) and localized study data from smaller geographical areas in the US and Western Europe because national surveillance data tended to report only overall syphilis trends while localized study data tended to report disparities (HIV, race, and age) in syphilis trends in addition to overall trends.

### Review of syphilis trend studies conducted in the US

#### Overall syphilis trend data in the US

**Fig 2:**
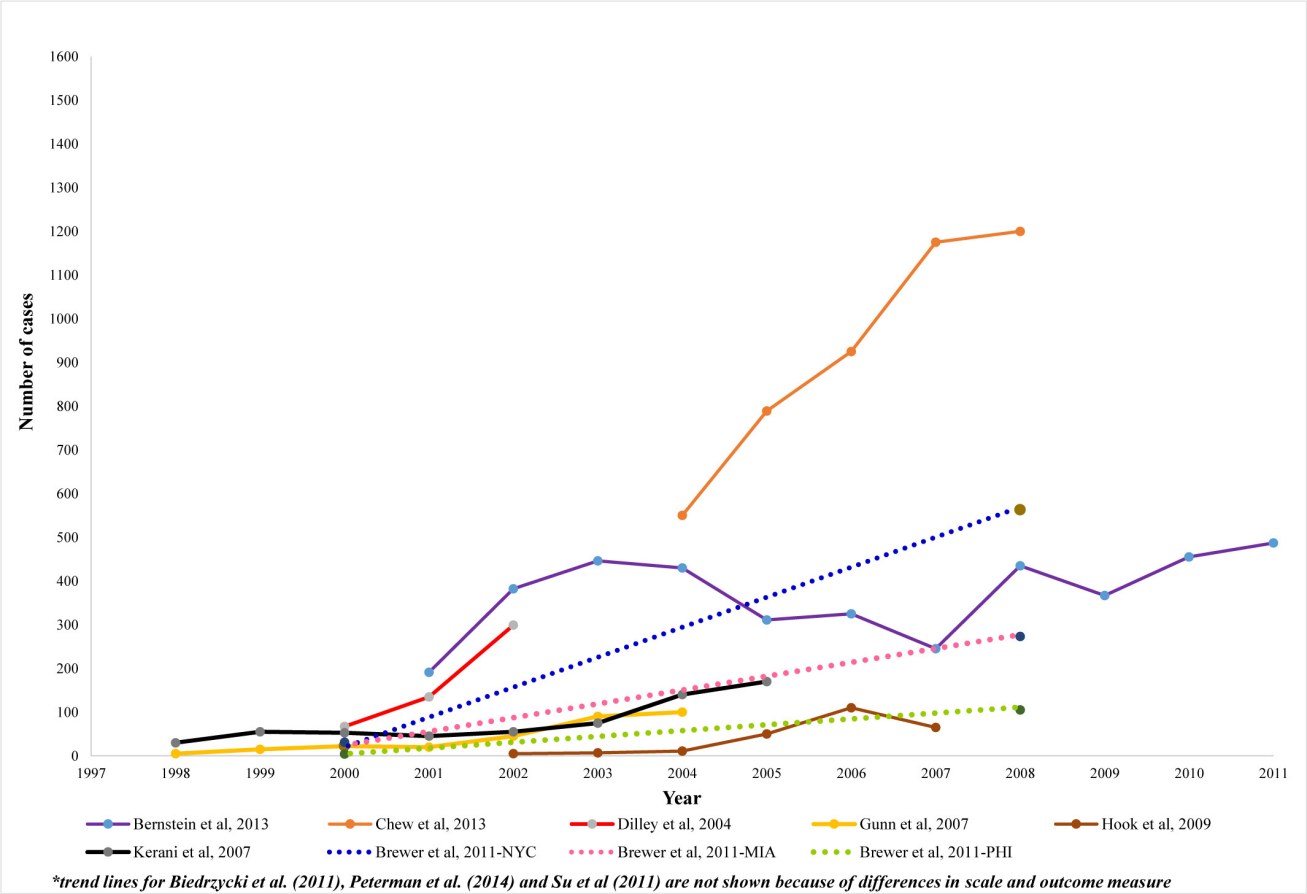
Syphilis trend studies in the United States: annual number of syphilis cases among MSM, 2004–2011.

Overall, the data from the studies conducted in the US document an increase in syphilis cases over time (Fig 2). Among most studies that commenced data collection between 1998 and 2000, syphilis cases were at their lowest during this period, but showed an increase from 2000 and beyond. Six studies reported a consistent increase (upward trend without a decline at any time point) over the period for which data were available [19–21, 25–27]. Gunn and colleagues (2007) examined syphilis cases among MSM in San Diego between 1998 and 2004 and reported a consistent increase [21]. The number of syphilis cases among MSM in the Gunn et al. study increased from less than 10 cases in 1998 to approximately 100 cases in 2004. Chew and colleagues (2013) also reported a consistent increase in the number of syphilis cases among MSM in California between 2004 and 2008 [19]. Su et al. 2011 used syphilis case data from 27 states and reported an increase among MSM from 3.4/100,000 males in 2005 to 4.0/100,000 males in 2008 [26]. Peterman and colleagues (2014) used national syphilis case data that was reported to the CDC from all states and the District of Columbia to examine syphilis trends and reported a consistent increase in syphilis cases among MSM from approximately 6,000 cases in 2006 to 14,000 cases in 2013 [27].

Four studies reported an overall increase in syphilis over time, despite intermittent periods of declining reported syphilis diagnoses [18, 22–24]. Kerani et al. (2007) assessed syphilis trends between 1998 and 2005 among MSM in King County, Washington [23]. Syphilis diagnoses remained stable between 1998 and 2001, and consistently increased from approximately 45 in 2001 to 180 diagnoses in 2005 [23]. Hook and colleagues (2009) assessed syphilis trends between 2002 and 2007 among MSM in Jefferson County, Alabama [22]. They documented an overall increase in syphilis cases during the study period, increasing from less than 5 cases in 2002 to greater than 60 cases in 2007. Two studies conducted tests of significance of syphilis trends [25, 26]. Brewer et al. (2011) conducted tests of significance in their trend analysis of surveillance data and concluded that the increase in syphilis prevalence from 2000 to 2008 among all three study sites were statistically significant (p<0.05) [25]. The number of syphilis cases significantly increased from 20 cases in 2000 to 440 cases in 2008 in New York City; 29 cases in 2000 to 223 cases in 2008 in Miami-Fort Lauderdale; and from 2 cases in 2000 to 70 cases in 2008 in Philadelphia. These findings were consistent with those of Su and colleagues (2011), who used syphilis case reports from 27 states from 2005 to 2008 and determined that the increase in syphilis case rates over this time period was statistically significant (p<0.05) [26]. Overall, as Fig 2 demonstrates, syphilis cases among MSM in the US have increased from their lowest prevalence in 1998 in a number of diverse geographic settings.

#### HIV infection and syphilis trend data in the US

Given the reports of syphilis increases among HIV-positive MSM, we assessed whether trends in syphilis differed by HIV status. Kerani et al. (2007) [23] and Biedrzycki et al. (2011) [24] examined the association between syphilis trends and HIV among MSM. Kerani and colleagues (2007) used surveillance data from King County, Washington, to compare trends in the rate of primary and secondary syphilis among MSM by HIV status [23]. The authors found that the rate of syphilis among HIV-negative MSM increased from 41 cases per 100,000 MSM in 1999 to approximately 200 cases per 100,000 MSM in 2005. The increase among HIV-positive MSM was significantly higher over the same time period, increasing from 45 cases per 100,000 MSM to 1,969 cases per 100,000 MSM. Biedrzycki and colleagues (2011) used surveillance data between 1999 and 2008 from the Wisconsin Department of Public Health to investigate the relationship between syphilis trends and HIV infection among young Black MSM in Milwaukee County, Wisconsin [24]. The authors observed that an increase in syphilis cases was indicative of increased risk behaviors and high-risk sexual networks of young Black MSM in Milwaukee County, and hypothesized that these factors also likely facilitated an increase in HIV cases in this population. In summary, data from these two studies suggest that the increases in syphilis trends may be more pronounced among HIV-positive MSM and that increased syphilis transmission may be accompanied by increased HIV transmission among MSM and vice versa.

#### Race and syphilis trend data in the US

This systematic review also evaluated racial differences in syphilis trends (Table 1). Su et al. (2011) [26], McFarland et al. (2004) [37], Patton et al. (2014) [2], and Fuqua et al. (2015) [38] investigated syphilis trends by race among MSM. McFarland and colleagues used surveillance data to compare trends in primary and secondary syphilis cases between Asian and Pacific Islander (API) MSM and White MSM in San Francisco between 1999 and 2002. Their findings showed racial differences in syphilis trends over time. In 1999, the prevalence of syphilis among API MSM and among White MSM were very similar, approximately 100 cases per 100,000 MSM. However, between 1999 and 2002, the magnitude of the increase in syphilis prevalence among API MSM was greater than White MSM. By 2002, the syphilis prevalence among API MSM was estimated at 1600 cases per 100,000 compared to approximately 800 cases per 100,000 among White MSM, despite the similar rates for these two groups in 1999 [37]. Su and colleagues used syphilis case reports from 27 states to examine racial disparities in syphilis trends among Black, Hispanic, and White MSM between 2005 and 2008 [26]. Their study findings showed significant disparities in syphilis trends. There was a significant increase in syphilis case rates between Black and White MSM (p<0.001) and between Hispanic and White MSM (p<0.001) from 2005 to 2008 [26]. The absolute increase in syphilis rates among Black and Hispanic MSM was 8.0 and 2.4 times the absolute increase in syphilis rate among White MSM respectively. After adjusting for region and age, the syphilis case rates increased by 74% among Black MSM, 61% among Hispanic MSM, and 34% among White MSM during the study period. Fuqua and colleagues used data from 2004 to 2011 from the San Francisco Municipal STD Control Department to examine trends in syphilis cases among Black MSM [38]. Their study concluded that there was an overall increase in syphilis cases among Black MSM in San Francisco during the study period despite intermittent periods of decline in syphilis cases [38].

Finally, Patton and colleagues used surveillance data from 34 states and the District of Columbia to determine racial differences in syphilis trends among MSM between 2009 and 2012 [2]. Overall the proportion of primary and secondary cases attributed to MSM increased from 77.0% in 2009 to 83.9% in 2012 but the greatest percentage increase occurred among Hispanic MSM (53.4%, from 1,291 in 2009 to 1,980 in 2012). There was a 38.1% increase (2,449 to 3,381) among White MSM and a 21.2% increase (2,267 to 2,747) among Black MSM [2]. The absolute number of syphilis cases among Black MSM was greater than the number of syphilis cases among Hispanic MSM and similar to the number of syphilis cases among White MSM [2], even though 71.4% of all MSM in the US are White, 15.9% are Hispanic and 8.9% are Black [39]. In summary, these data present evidence for widening racial disparities in syphilis trends among MSM in the US with racial minority (Black, Hispanic, and API) MSM bearing a disproportionate burden.

#### Age and syphilis trend data in the US

We examined syphilis trends by age among MSM. Brewer and colleagues assessed syphilis trends by age (15–19 years, 20–24 years, and ≥ 25 years) among MSM in three cities (New York City, Philadelphia, and Miami-Fort Lauderdale) [25]. Using surveillance data from these three cities, they demonstrated significant increases in syphilis cases from 2000 to 2008 among MSM between 20 and 24 years and MSM ≥ 25 years in all three cities (p<0.05). There was a significant increase in syphilis cases among MSM between 15 and 19 years in New York City only. Consistent with these findings, Su and colleagues, using data from 27 states, observed that the greatest absolute increase in syphilis rates between 2005 and 2008 occurred among MSM between 20 and 24 years and MSM between 25 and 29 years of age [26]. To a lesser extent, there were absolute increases in syphilis rates among MSM between 15 and 19 years, 30 and 34 years, and 40 and 44 years while there were absolute decreases among MSM between 35 and 39 years and MSM ≥ 45 years. Lastly, Patton and colleagues examined data from 2009 to 2012 from MSM in 34 states and the District of Columbia and showed that the greatest percentage increases by age group occurred among MSM aged between 25 and 29 years (53.2%, 1,073–1,644) [2]. These findings suggest that young MSM, especially MSM between 20 and 29 years, may account for the greatest increases in syphilis trends among all MSM in the US.

### Review of studies conducted in Western Europe

#### Overall syphilis trend data in Western Europe

**Fig 3:**
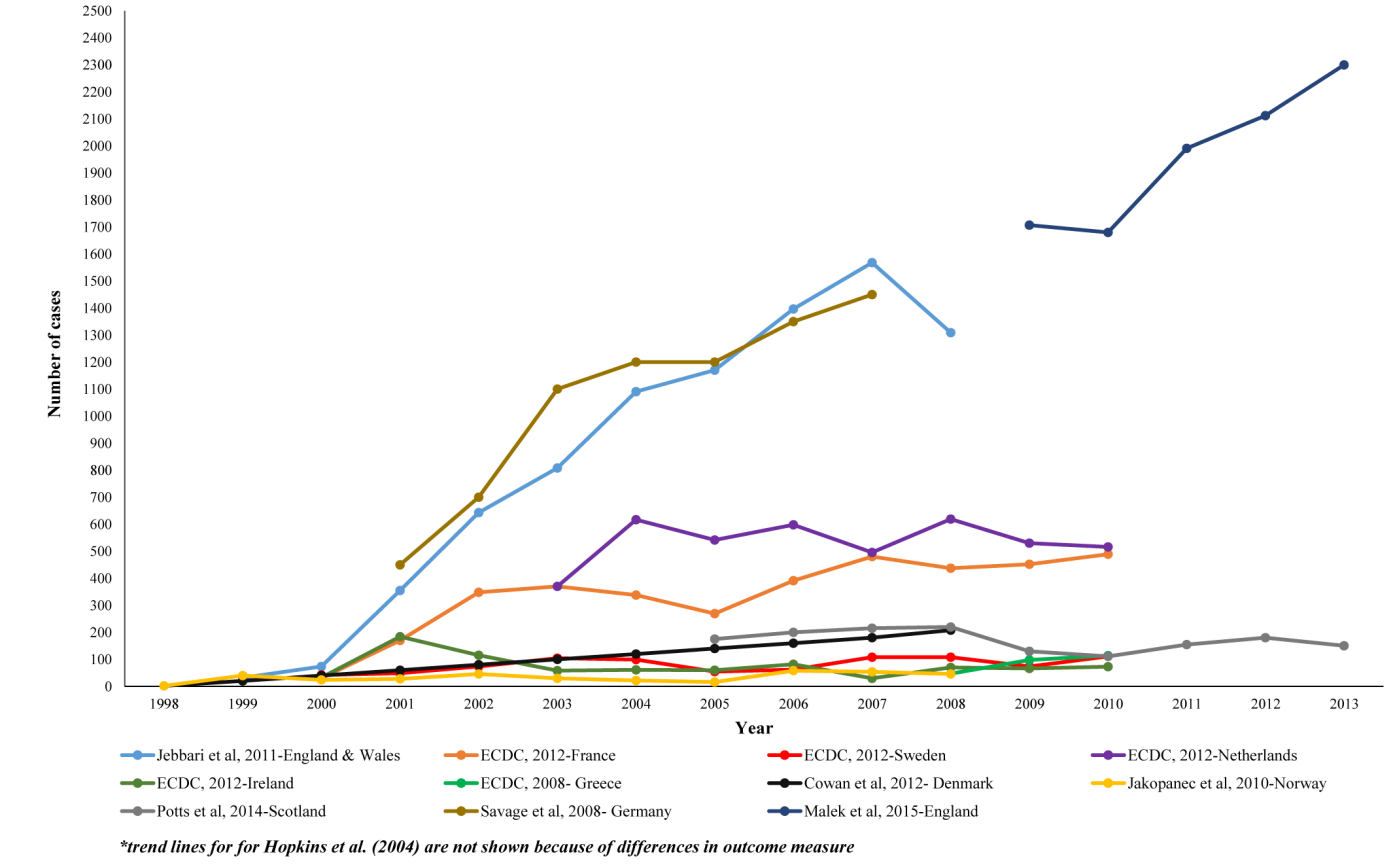
Syphilis trend studies in Western Europe: annual number of syphilis cases among MSM, 2004–2015.

Fig 3 illustrates trends in reported syphilis cases among MSM in Western Europe from 1998 for the studies and surveillance reports included in this review. Similar to syphilis trends among MSM in the US, the trend lines suggest an increase in syphilis prevalence over time among MSM in Western Europe. In all the studies, syphilis cases among MSM in Western Europe were lowest between 1998 and 2000 and have increased since. Two studies presenting syphilis surveillance data from Greece and Germany showed a consistent increase in syphilis cases [35]. National surveillance data from Germany showed a consistent increase in syphilis cases among MSM, increasing from approximately 400 cases in 2001 to 1500 cases in 2007 while national surveillance data from Greece showed an increase from 47 cases in 2008 to 114 cases in 2010 [35]. The remaining studies showed an overall increase despite intermittent periods of decline [28]. Malek et al (2015) reported an increase in the number of syphilis diagnosis among MSM in England from 1707 syphilis cases in 2009 to 2300 cases in 2013 [29]. Using surveillance data, Jebbari et al (2011) reported an increase in the number of syphilis cases among MSM in England and Wales from 808 cases in 2003 to 1568 cases in 2007 [28]. In France, national case surveillance data show an increase in the number of syphilis cases among MSM from 30 cases in 2000 to 489 cases in 2010 [32]. Hopkins and colleagues reported an increase in syphilis case rates from 5.1/100,000 in 1998 to 137/100,000 in 2003. Another study conducted in Sweden demonstrated an increase in the number of syphilis cases among MSM from 42 cases in 1998 to approximately 111 cases in 2010 [32]. Potts and colleagues (2014) examined syphilis trend data among MSM in Scotland from 2005 to 2013 [30]. Their study findings showed an uneven syphilis trend during the study period. There was a consistent increase in national syphilis cases in Scotland between 2005 and 2008, followed by a decline in cases 2009 and 2010, then a steady increase in 2011 and 2012, and a decline in 2013. Finally, an analysis of national surveillance data from the Netherlands also demonstrated an overall increase in syphilis cases among MSM from approximately 350 cases to greater than 516 cases between 2003 and 2010 [32, 34].

#### HIV infection and syphilis trend data in Western Europe

Five studies examined the association between syphilis and HIV status among MSM [29, 31, 33, 34, 40]. Hopkins et al. (2004) examined differences in rates of syphilis by HIV status among MSM attending a health center in Ireland [31]. Syphilis rates increased from 0 per 100,000 in 1999 to 1,111 per 100,000 in 2003 among HIV-positive MSM in this study. This rate was more than 10 times as great in HIV-positive MSM versus HIV negative MSM over the same period [31]. Jakopenac and colleagues (2010) utilized case surveillance data to examine HIV co-infection among MSM with newly diagnosed syphilis cases in Norway between 1999 and 2008 (p < .009) [33]. The authors reported a decrease in HIV/syphilis co-infection from 1999 to 2001, a sharp increase in cases in 2002, and a decrease in 2003, the year that accounted for the lowest number of cases recorded during the study period. However, there was a resurgence in the number of HIV/syphilis co-infection cases among MSM from 2003 to 2008, with a six-fold increase in HIV/syphilis co-infection cases between 2003 and 2008. The authors concluded that the proportion of MSM co-infected with HIV significantly increased over time (p = 0.009), peaking at 39% in 2008 [33].

Marcus and colleagues (2005) analyzed the relation between HIV and syphilis trends among MSM in Germany [40]. Their findings indicated that between 2001 and 2003, the proportion of syphilis cases among MSM with coincident HIV at diagnosis (syphilis and HIV diagnosed within a time period of plus or minus three months) increased from 5% to 7%. Cowan and colleagues (2012) examined syphilis cases among Danish MSM by HIV status between 1998 and 2010 [34]. Their study showed a greater number of syphilis diagnoses among HIV-positive MSM than among HIV-negative MSM throughout most of the study period [34]. Lastly, Malek and colleagues (2015) assessed syphilis trends among HIV-positive MSM in England between 2009 and 2013 [29]. The authors found that between 2009 and 2013, the odds of being diagnosed with syphilis increased significantly from 2.71 to 4.05 in HIV-positive MSM relative to HIV-negative/undiagnosed MSM. There was also a consistent increase in the proportion of syphilis cases among HIV-positive MSM, increasing from 25% in 2009 to approximately 38% in 2013 [29]. These studies highlight the association between HIV infection and syphilis trends among MSM.

#### Race, age, and syphilis trend data in Western Europe

Very few studies conducted in Western Europe presented differences in syphilis trends by race and age. However, Jebbari and colleagues (2011) noted that the demographic profile of the increasing trends in syphilis in England and Wales was comprised predominantly of White MSM [28]. The study by Koedijk et al. (2014), using national surveillance data from the Netherlands, did not demonstrate any age differences in syphilis trends among MSM, rather showing a decline in syphilis cases among both younger MSM (15–24 years) and older MSM (25 years and above) [36].

## Discussion

We examined trends in reported syphilis cases from 1998 and onwards in order to advance our understanding of syphilis disease patterns over time among MSM in the US and Western Europe. The findings of our review showed that syphilis cases increased overall among MSM in the US and Western Europe from 1998. We also found disparities in syphilis trends by HIV status, race, and age among MSM. Among MSM in the US and Western Europe, increases in syphilis cases over time were greater among HIV-positive MSM than among HIV-negative MSM with a corresponding increase in the number of HIV/syphilis co-infections [23, 24, 29, 31, 33, 34, 40]. Racial minority MSM (Black, Hispanic, and API) accounted for a greater increase in the number and rates of syphilis cases over time compared to White MSM in the US [2, 37, 38]. By age group, the greatest increases in the number and rate of syphilis cases over time in the US occurred among MSM between 20 and 29 years [2, 25, 26, 38]. Few studies examined racial and age disparities in syphilis trends among MSM in Western Europe [28, 36]. In Western Europe, because White MSM comprise the majority of MSM, they accounted for the greatest increases in syphilis trends. One study in this review reported a decrease in syphilis cases over time among younger (15–24 years) and older (≥25 years) MSM and there was no study included in this review that examined racial disparities in syphilis trends among MSM in Western Europe.

The reason for the increase in syphilis cases among MSM in the US and Western Europe is unknown but several factors could be contributing to the increases. Several studies suggest that condom use is decreasing among MSM [8, 41, 42]. The reduction in condom use is due in part to increases in sero-adaptive behaviors like sero-sorting (selecting a sexual partner who reports the same HIV status) and sero-positioning (choosing a sexual position depending on the HIV status of one’s partner) among MSM in the US and Western Europe [43–46]. These behaviors can reduce the risk of transmitting HIV among sexually active MSM, however they do not offer any protection against syphilis and other STIs and may in fact inadvertently increase the risk of syphilis transmission in the presence of CAS [47]. The use of the Internet and Internet-based mobile applications to meet sexual partners commenced in the late 1990s and is now prevalent among MSM in the US and Western Europe [48–50]. MSM who engage in risky sexual behaviors may use the Internet and Internet-based mobile applications to facilitate casual and anonymous sexual contacts, which can drive the transmission of syphilis and other STIs. Some syphilis outbreaks in the US and Western Europe among MSM have been traced to casual and anonymous sexual encounters facilitated by the Internet [51, 52].

Syndemics may also be contributing to the increases in syphilis cases over time among MSM in the US and Western Europe [3, 53]. A syndemic refers to the concentration within a specific population of multiple co-occurring epidemics or conditions that can amplify the negative impact of one or more other health problems [54]. For example, sub-populations of MSM with high prevalence of substance abuse also report high prevalence of risky sexual behaviors such as multiple and anonymous sex partners and CAS [3] which can facilitate syphilis transmission [55]. HIV increases the risk of syphilis transmission [56]. High HIV-prevalence among sub-populations of MSM can also facilitate syphilis transmission since HIV-positive persons with syphilis have deeper ulcers that do not heal quickly and may also be at a higher risk of treatment failure [7, 56].

The increase in syphilis cases over time observed in this review could also be explained by expanded syphilis testing initiatives that target MSM and recent guidelines that recommend syphilis among sexually active MSM. Furthermore, improved surveillance and reporting systems in the US and Western Europe could have contributed the increase in syphilis cases over time. This is important to note because many of the studies included in this review utilized surveillance data. Understanding the different healthcare systems in the US and Western Europe may further aid our understanding of the syphilis trends in both geographic areas. In Western Europe, access to STI consultation services including prevention resources and treatment is almost universal and usually at no cost [57]. In contrast, in the US, STI prevention and treatment is not universal and some populations experience barriers to accessing STI care [58]. The similar increase in syphilis trends in both geographical areas despite different sexual healthcare systems might suggest that these increases are true increases in syphilis cases and may not only be due to improved syphilis surveillance and access to testing services among MSM in the US and Western Europe.

A number of factors may explain disparities in syphilis trends by HIV status, race, and age. MSM, especially HIV-positive MSM, may view a diagnosis of syphilis as curable and less severe than HIV infection and may therefore be less concerned about acquiring syphilis [59]. Social factors also affect syphilis transmission, especially among racial minority populations [3, 60]. Structural-level factors such as poverty, social marginalization, lower educational level and an inability to access healthcare services, which are more prevalent among racial minorities, can result in barriers to prompt diagnosis and treatment for STIs [3, 60]. Collectively, these factors can impact the prevalence of STIs, particularly within densely connected sexual networks of MSM. Racial assortative sexual partnerships within these populations can also foster the transmission and spread of syphilis among racial minority MSM. Expanded syphilis testing and surveillance programs that target racial minority MSM such as Black and Hispanic MSM can result in the identification of a greater number of syphilis infections and therefore account for the higher numbers of syphilis diagnoses in these groups of MSM. Lastly, complacency regarding sexual risk and normative attitudes that favor risk behaviors, common among young MSM [61], can explain age-related disparities in syphilis case trends among MSM in the US.

Public health strategies to reverse the increases in reported syphilis among MSM are warranted, particularly as majority of the studies presented trends data that demonstrated an increase in primary and secondary syphilis. Syphilis transmission occurs with primary and secondary syphilis infection thus screening and treatment are vital to mitigate transmission. Possible avenues for further exploration include raising awareness among providers to increase prompt identification and treatment of syphilis in order to interrupt continued transmission of syphilis. Timely notification of all sexual partners to offer screening and treatment is also critical to syphilis control. Removing barriers to syphilis testing among MSM by expanding access to syphilis testing in non-traditional sites like community-based organizations, especially those that serve MSM, and concurrently screening for syphilis at every HIV test are other avenues that ensure that all sexually active MSM get screened for syphilis.

Syphilis screening programs that target high-risk MSM such as MSM who report multiple partners, sero-adaptive behaviors, illicit drug use and repeat syphilis infections may be beneficial in mitigating ongoing syphilis transmission. The CDC recommends syphilis testing every 3 to 6 months among at-risk MSM [62]. Allowing MSM to request a syphilis test online and retrieve their results online is another strategy that can remove barriers to widespread and regular syphilis testing [63]. This approach was successful in expanding access to syphilis testing and eliminating delays in obtaining syphilis tests in San Francisco [63]. Given the syphilis risk among MSM and suboptimal screening rates among MSM [64], ensuring that syphilis screening is a routine part of sexual and HIV care for all MSM is critical. System-level efforts to improve syphilis screening among MSM may be an effective approach to prevention. Standing screening protocols and electronic medical records prompts that remind providers to routinely assess sexual risk among sexually-active MSM and offer syphilis screening has been used with success in identifying early syphilis infection among high-risk MSM [65, 66].

Given the syphilis HIV synergy, healthcare providers should ensure that HIV-positive MSM get tested for syphilis as frequently as recommended in order to avoid ongoing transmission. These clinical encounters should promote consistent condom use and safer sex behaviors, educate MSM on the effect of syphilis on HIV risk and disease, emphasize the risk of syphilis transmission associated with sero-adaptive behaviors and oral sex, and initiate immediate treatment of MSM and their sexual contacts when necessary. There is also a risk of syphilis among MSM on pre-exposure prophylaxis (PrEP) [67], hence STI testing must be emphasized and these MSM should be tested for syphilis and other bacterial STIs every six months, per current recommendations for MSM [68].

Culturally appropriate evidence-based public health education programs that are geared towards improving community-level knowledge, sexual health promotion, increasing condom use, and syphilis testing are effective. Preliminary results from syphilis public health education programs in eight US cities showed an increase in syphilis awareness and testing among MSM [69]. Finally, establishing partnerships with MSM community organizations can further raise awareness and perception about syphilis infections and testing within the MSM population in the US and Western Europe.

There are some limitations to this systematic review of the literature. Articles that were not published in English and unpublished studies (e.g., dissertations, conference abstracts and presentations) were not included in this review. This review was a qualitative description of studies on syphilis trends among MSM and not a meta-analysis so we could not calculate and compare the effect sizes of the various studies. The findings of the studies in this review were based on convenience samples and case surveillance data. Syphilis case notification is mandatory in some countries in Western Europe and voluntary in others [32]. Some other countries in Western Europe do not collect syphilis surveillance data on MSM [32, 35]. These differences in data sources and syphilis case notification could have introduced bias in the comparison of syphilis trends.

Nearly all data included in this review were reported as syphilis case counts and not rates, which limits the true estimation of risk of syphilis in an epidemiologic sense. Estimating the population size of MSM is challenging due to biases in the various data sources [70, 71] and changes in the size of the population of persons who identify as MSM or report a male sex partner, thus making it difficult to identify an appropriate population at risk for syphilis in MSM-specific analyses. Before 2005, the number of syphilis cases in MSM in the US had to be estimated because data on MSM status were unavailable to allow case counting among MSM. This could have affected our interpretation of syphilis trends among MSM in the US in this review. However, a recent study that estimated the MSM population in the US can be used to estimate syphilis rates in the future [72]. Age cohort effects could have affected our interpretation of age disparities in syphilis trends. Some trend studies from Western Europe reported on primary and secondary syphilis while others reported on primary, secondary and latent syphilis. This inconsistency in syphilis reporting could have biased our interpretation of trends in Western Europe. Few studies from Western Europe examined trends in syphilis among MSM populations most affected by syphilis such as HIV-positive persons, racial minorities, and younger persons. The paucity of these studies limits any conclusion about syphilis trends in these populations. Studies in this review were restricted to MSM in the US and Western Europe and may not be representative of syphilis trends among MSM globally.

## Conclusion

This review examined trends in reported syphilis cases counts and rates among MSM in the US and Western Europe generally and stratified by HIV status, race, and age. Our findings indicate that the number of syphilis cases and rates among MSM in the US and Western Europe have increased since 1998. Continuous surveillance to monitor, identify, and address syphilis clusters among MSM, sexual risk assessment and counseling during clinical encounters, consistent condom use, frequent syphilis testing and prompt treatment of index MSM cases and their sexual contacts are essential to syphilis control. Innovative evidence-based syphilis prevention interventions for all MSM, but especially HIV-positive MSM in the US and Western Europe and racial minority and younger MSM in the US are needed to reverse the syphilis trends in these subpopulations. Successful implementation of these measures is essential to addressing syphilis among MSM.

## Supporting Information

S1 Checklist PRISMA 2009 Checklist. (DOC)
